# Plasma oxidative stress in reproduction of two eusocial African mole-rat species, the naked mole-rat and the Damaraland mole-rat

**DOI:** 10.1186/s12983-021-00430-z

**Published:** 2021-09-17

**Authors:** Paul Juan Jacobs, Daniel William Hart, Nigel Charles Bennett

**Affiliations:** Department of Zoology and Entomology, Mammal Research Institute, University of Pretoria, Pretoria, 0002 South Africa

**Keywords:** Oxidative stress, Mole-rat, Redox balance, Total oxidant status, Total antioxidant capacity, Reproduction

## Abstract

One of the most prominent life-history trade-offs involves the cost of reproduction. Oxidative stress has been proposed to be involved in this trade-off and has been associated with reduced life span. There is currently an unclear relationship between oxidative cost and the reproduction-longevity trade-off. The current study, using a non-lethal and minimally invasive (only a single blood sample and no euthanasia) method, investigated whether an oxidative cost (oxidative stress) to reproduction would be apparent in two long-lived eusocial mole-rats, the naked mole-rat (NMR), *Heterocephalus glaber*, and the Damaraland mole-rat (DMR), *Fukomys damarensis*, where breeding colony members live longer than non-breeder conspecifics. We measured the direct redox balance in plasma by measuring the oxidative stress index (OSI) based on the ratio of total oxidant status and total antioxidant activity in breeders and non-breeders of both sexes, in the two species. NMR had significantly higher OSI between breeders and non-breeders of each sex, whereas DMR showed no significant differences except for total antioxidant capacity (TAC). The mode of reproductive suppression and the degree of reproductive investment in NMR may explain to some degree the redox balance difference between breeders and non-breeders. DMR show minimal physiological changes between breeders and non-breeders except for the mode of reproduction, which may explain some variations in TAC and TOS values, but similar OSI between breeders and non-breeders.

**Supplementary Information:**

The online version contains supplementary material available at 10.1186/s12983-021-00430-z.

## Introduction

Life history theory addresses the trade-offs that shape animal investment patterns between reproduction, somatic maintenance and longevity [1–3]. One such life-history trade-off may be mediated by free radical production and oxidative stress [1]. Oxidative stress arises when reactive oxygen species (ROS) production, which damages proteins, lipids, and DNA, exceeds the antioxidants capacity and repair mechanisms to prevent or mitigate ROS damage [4–6]. Despite the adverse effects associated with increased ROS levels, ROS as a biological molecule is essential to cellular signalling [7], to inflammation response [8], altering glucose uptake and metabolism [9], immune response [10], allowing for the preparation to deal with hypoxic stress [11] and osmoprotective signalling [12].

Reproduction and the subsequent production and development of offspring have been linked to compromised survival, with prolific reproduction associated with a significantly shorter life span [13–16]. The physiological costs of reproduction can impair the functionality of other physiological processes, with these costs resulting from the reduction of resources available for self-maintenance, but there may also be direct effects of the process of reproduction itself [17, 18]. In the past, oxidative stress was suspected to impart a cost to reproduction [6, 19–21]. More recent studies on the oxidative cost of reproduction have either found no link or weak support for this life history trade-off hypothesis [22]. Interestingly, evidence suggests that reproducing females have chronically lower oxidative damage in some species and subsequently lower oxidative stress than non-reproducing individuals [3, 23]. This finding has subsequently been proposed as the oxidative shielding hypothesis, where reproducing females may resort to pre-emptive reductions in oxidative damage and/or stress during sensitive periods of reproduction (e.g. gestation, lactation). Possible reasons for these varied responses in past studies are shortcomings within the experimental setup [22–24]. These shortcomings include artificially manipulating reproductive effort, where individuals were forced not to reproduce or required an extra cost to the reproductive effort, which may not occur naturally [23]. An example includes brood size manipulation, where brood size was increased to infer an increased cost to reproductive effort relative to controls [25]. Furthermore, individuals can set their limits on reproductive effort, with several characteristics, such as body condition, hormonal profile, behaviour, food availability and glucocorticoid concentration, all of which can influence reproduction [23, 26–30], which is likely to vary between and within species.

This study proposes that long-lived eusocial mole-rats, namely the naked mole-rat (NMR), *Heterochepahlus glaber*, [31, 32] and the Damaraland mole-rat (DMR), *Fukomys damarensis* [33–35]*,* are perfect model species to investigate whether an oxidative cost to reproduction is evident, as reproduction is naturally controlled within the species, with no experimental manipulation required for individuals who do not breed. In species that exhibit an eusocial behaviour, a few dominant animals monopolise reproduction, with one breeding female (BF) and one to three of the larger males (breeding males-BMs) are responsible for reproduction [36]. Apart from reproductive division of labour, cooperative care of the young and overlap of generations, the lifetime reproductive success (LRS) among members of both NMR and DMR colonies squarely places these two species on the eusocial end of the eusociality spectrum (see Sherman et al. [37]). The remaining colony members (non-breeding females—NBFs and non-breeding males—NBMs) are reproductively quiescent [36], where both NBFs and NBMs can reproduce, but are naturally reproductively suppressed through both behavioural and physiological mechanisms [36, 38–42]. The consequences of this suppression are that reproductive hormones, such as oestrogen, progesterone and testosterone, are significantly higher in the breeding colony members than those of non-breeding colony members [43]. The non-breeding colony members’ reproductive hormones and gonad development are comparable to juvenile (sexually immature) anovulatory colony members [36]. As with eusocial insects, female reproducing members in NMRs and DMRs as well as Ansells (*Fukomys anselli*) and Giant (*Fukomys mechowii*) mole-rats colonies are often the longest-lived [34, 41, 44–46]. However, to date, due to a lack of empirical data pertaining to LRS for *F. anselli* and *F. mechowii* they do not fit the stricter conditions for the claim of eusociality according to the definition of Sherman et al. [37], which is bimodality, resulting in a high reproductive skew among breeding females and/or males where maximum lifetime fecundity of breeders versus non-breeders (helpers) is far greater than that of female breeders versus helpers in cooperatively breeding vertebrate societies. In this definition the proportion of non-breeding animals obtaining reproductive status during their lifetime can be used as a stricter measure of eusociality when compared to the classic definition used by Michener back in the 1960s [47], which simply having overlapping generations, cooperative brood care, and reproductive division of labor.

The method and the subsequent physiological consequence of reproductive suppression differ between the NMR and DMR. Within NMRs, which spontaneously ovulate, strong physiological reproductive suppression occurs, resulting from neuroendocrine changes that lead to halted follicle development, stunted at the primordial stage, and anovulation in females and reduced spermatogenesis in males [35, 48]. Interestingly, hyperprolactinemia and raised plasma prolactin concentrations have been observed to be the possible neuroendocrine mechanism that causes physiological reproductive suppression in non-breeding NMR colony members [49, 50]. In NMRs, non-breeding colony members were found to possess similar plasma prolactin levels compared to BFs, even those that were pregnant and lactating [49]. Contrastingly, in the same study, over 85% of DMRs non-breeding individuals had undetectable prolactin values, whereas breeders demonstrated expected variance in prolactin concentrations due to normal reproductive function [49]. In DMRs, which are induced ovulators [51], a behavioural mode of suppression is favoured, which is brought about by subordinates avoiding breeding with related individuals in a colony (incest avoidance) and/or the non-breeding colony members being the object of aggression and having their reproductive behaviour interrupted by the breeding individuals [51, 52]. In addition to the behavioural mode of reproductive suppression, physiological suppression occurs through the mechanism of reduced luteinising hormone (LH) and a reduced basal level of gonadotropin-releasing hormone, with the resulting reduced LH stimulation preventing ovulation in the presence of the BF [42, 53]. Consequently, follicle development is arrested at the Graafian follicle stage in DMR NBFs, but spermatogenesis occurs in DMR NBM [35, 48]. The introduction of a unrelated male into a queenless colony (reproductively quiescent) allowed one NBF to become reproductively active [54]. This suggests that both factors: an unrelated BM and the absence of the BF are needed to initiate reproduction in a DMR NBF [42, 54]. Unlike in NMRs physiological reproductive suppression is not dependent on prolactin in DMRs as plasma prolactin levels are undetectable under normal, non-lactating conditions in non-breeding colony members [49]. It can therefore be hypothesised that non-breeding DMRs invest more into reproduction than non-breeding NMRs due to differences in energy investments in follicle development and spermatogenesis.

The current study used a non-lethal and minimally invasive (only a single blood sample and no euthanasia) method to investigate the direct plasma redox balance of both eusocial mole-rat species (NMR and DMR) across sex and reproductive status (breeding and non-breeding). Generally, redox balance studies involve multiple markers and/or tissue samples resulting in destructive sampling (euthanasia of the animals and tissue harvesting), wherein longitudinal studies or studies on rare animals are not feasible, and blood sampling is the only option [55]. Furthermore, the collection of large plasma volumes from small animals are generally unfeasible and unethical. Previous studies investigating direct redox balance in animals measured the ratio between glutathione (GSH) and glutathione disulfide (GSSG). This is generally performed in tissues and requires a large volume of sample, as GSH measured in plasma is generally below the detection limit. Furthermore, plasma measurements of GSH/GSSG have been observed to lead to erroneous and possibly misleading measurements due to overestimation of GSSG [56, 57]. Consequently, the use of the oxidative stress index (OSI) from total oxidant status (TOS) and total antioxidant status/capacity (TAS/TAC) may be a helpful alternative in measuring oxidative stress in small animals that cannot be euthanised. The OSI index does not require large plasma volumes, ideal for smaller animals, as well as not resorting to destructive sampling. This allows for the direct measurement of redox balance in animals in longitudinal and rare animals, like breeding mole-rat individuals.

Using the only two eusocial mole-rat species as currently defined by the low lifetime reproductive success of non-breeding subordinates, we will attempt to confirm the actual effect of reproduction on fitness on mammals through the measurement of oxidative stress. Since both mole-rat species in this study are long-lived, subterranean, and are eusocial, we expect them to demonstrate similar oxidative stress patterns in reproduction.

## Methods

### Study species

Thirty-one NMRs of similar age (3–5 years old) (eight BFs, seven BMs, seven NBFs, eight NBMs) from among six captive colonies and 32 DMRs of similar age (3–5 years old) (eight BFs, eight BMs, eight NBFs, eight NBMs) from among 12 captive colonies from the University of Pretoria were used. All individuals were considered adults for this experiment as each individual, if not reproductively suppressed, would be able to reproduce (N.C. Bennett per comm). For both species, BFs were identified by the presence of prominent axillary and inguinal teats, well-developed external genitalia with a perforate vagina, and/or pregnancy-related changes in girth/body size. BMs were identified based on observations of copulation with the BFs (NMRs), while in DMRs a dark stain around the periphery of the mouth and bulging testes which project from abdominal pockets discern dominant breeders. Long-term observational records were used to confirm the identity of the socially dominant males and females. All animals have been a part of long-term (+ 20 years) monitoring and breeding projects at the University of Pretoria. Accurate age data was available (accuracy to 1 day) for non-breeding NMRs, while less accurate age data was available for the breeding NMR colony members (accuracy to 1 year). Likewise, only less accurate age data was available for the breeding and non-breeding DMR colony members (accuracy to 1 year). Breeders are generally found to be larger and older individuals [36]. Further details are given in the supplementary electronic material (Additional file 1: Tables S1 and S2).

### Animal housing

NMRs were kept in tunnel systems with several plastic chambers serving as food storage, toilet and sleeping areas and connected by acrylic glass tunnels. Nesting material consisted of wood shavings. DMRs were housed in their natal colonies in large plastic crates (1 m × 0.5 m × 0.5 m), with wood shavings and paper towelling provided as nesting material. Housing room temperatures ranged between 24.5 and 27 °C (DMRs) and 29–30 °C (NMRs), with relative humidity around 50–60% [58–60]. Animal rooms were maintained on a 12L:12D photoperiod. Photoperiod has not been shown to affect DMR and NMR behaviour [61]. Animals were fed on a variety of chopped vegetables and drank no free water.

### Blood sampling

For both NMRs and DMRs, blood samples were collected between 08h00 and 13h00 as follows: The animals were handheld and venous blood samples collected from the hindfoot or tail. Approximately 300–500 µl of blood was collected into heparinised micro-haematocrit tubes depending on the body mass of the animal. The blood was centrifuged at 1300 g and the resulting plasma decanted and stored at – 80 °C until further analysis (< 1 month). Only 1% of the total body mass of the individual of blood was allowed to be collected as defined by the University of Pretoria Animal Ethics Committee.

### Reagents

Unless otherwise stated, all chemicals and reagents used in this study were obtained from Merck (Pty) Ltd (Gauteng, South Africa).

### Total oxidant status (TOS) assay

Plasma TOS levels were measured through Erel’s method [62]. Briefly, this method is based on the oxidation of ferrous ion to ferric ion in the presence of various oxidative species. The oxidation reaction is enhanced by glycerol molecules, which are abundantly present in the reaction medium. The ferric ion makes a coloured complex with xylenol orange in an acidic medium. The colour intensity, measured spectrophotometrically, is related to the total amount of oxidant molecules that are present in the sample. The results are expressed in terms of micromole hydrogen peroxide equivalent per litre (μmol H_2_O_2_ equivalent/L). Samples were run in duplicate and only once per plate with a repeatability of *r* = 0.95. Intra-assay variability (%CV) was 9.8%.

### Total antioxidant capacity (TAC) assay

Plasma TAC levels were quantified using a commercially available kit (Antioxidant Assay Kit, Cayman Chemical Co., Ann Arbor, MI, USA) which measures the oxidation of ABTS (2,29-Azino-di- [3-ethybenzthiazoline sulphonate]) by metmyoglobin, which is inhibited by non-enzymatic antioxidants contained in the sample. Oxidised ABTS is measured by spectrophotometry at a wavelength of 750 nm. The capacity of antioxidants in the sample to inhibit oxidation of ABTS is compared with the capacity of known concentrations of Trolox, and the results are expressed as micromole Trolox equivalents per litre (μmol Trolox equivalents/L). Samples were run in duplicate and only once per plate with a repeatability of *r* = 0.99. Intra-assay variability (%CV) was 2.8%.

### Oxidative stress index (OSI)

Oxidative stress was determined by the TOS:TAC ratio, which represents the oxidative stress index (OSI) arbitrary unit, which was calculated as follows: OSI = [(TOS, µmol H_2_O_2_ equivalent/L)/(TAC, µmol Trolox equivalent/L)] * 100 [63].

### Statistical analysis

To determine the sample size and effect size, a power analysis with the program G-power, assuming an alpha value of 0.05 and a power threshold of 0.8, the analysis indicated that the sample size per group (BF, BM, NBM and NBF per species) is 12, with an effect size of 0.85. However, due to the difficulty of obtaining and breeding these animals, we believe the chosen sample size will be adequate to access our question and result in valid statistical results.

All statistical analyses were performed in R 3.5.2 [64] (R Development Core Team, 2018). Species were analysed separately. The normality of the response variables (OSI, TAC or TOS) for each species were determined using Shapiro Wilk tests (S–W). Homogeneity of all dependent variables was confirmed with a Levene’s test. Log-transformation was attempted to normalise all non-normal data. Normally distributed dependent variables were analysed using a linear mixed model (LMM), whereas non-normally distributed dependent variables were analysed by generalised linear mixed models (GLMM) fitted with gamma distributions and link-inverse or log function using the *lme4* package [65]. All models included colony as a random factor. Post-hoc comparisons were made using Tukey HSD pairwise comparisons. Each model contained OSI, TAC or TOS as the response variables and sex (male or female) and breeding status (breeding or non-breeding) as predictors, with a breeding status*sex interaction included. Body mass was included as a covariant in all models for both species. Pearson correlation tests between age and OSI, TAC or TOS were run for NMR NBM and NBF separately. A Benjamini–Hochberg correction was conducted using a False Discovery Rate of 0.2 (20%). We did not investigate age as an effect on OSI, TAC or TOS in DMR or BF and BM NMR due to a lack of definitive ages for each individual. Data are presented as mean ± standard error (s.e.m).

## Results

### Damaraland mole-rats

**Fig. 1:**
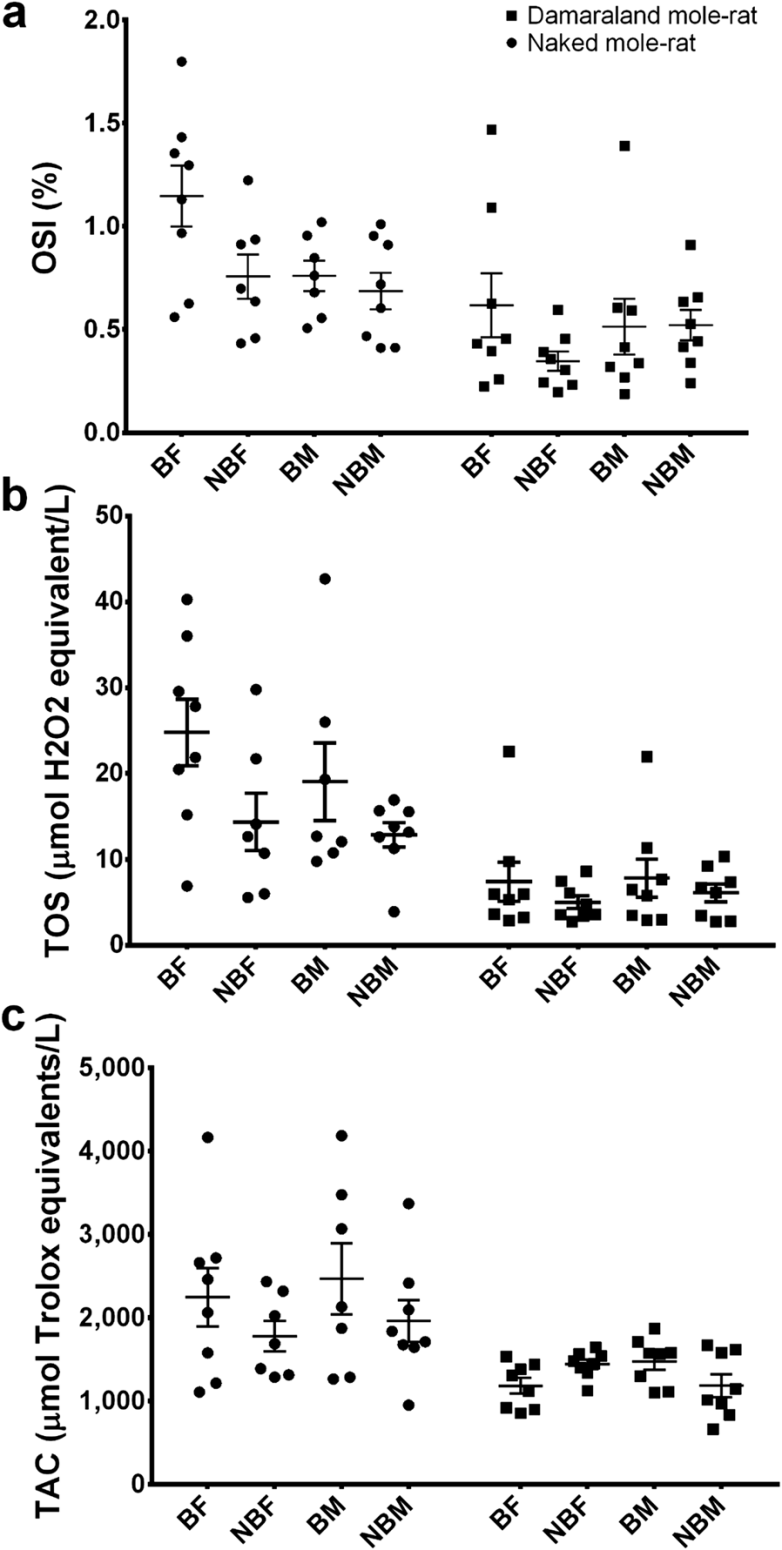
The **a** oxidative stress index (OSI-%) **b** total oxidant status (TOS-µmol H_2_O_2_ equivalent/L) **c** total antioxidant capacity (TAC-µmol Trolox equivalents/L) in Damaraland mole-rats and Naked mole-rats between breeding (BF) and non-breeding (NBF) females, and breeding (BM) and non-breeding males (NBM) males. The solid squares (■) represent Damaraland mole-rats and the solid circles (●) represent the naked mole-rats. Mean ± s.e.m

Both the breeding status (t =  − 1.69, *p* = 0.10) and sex (t =  − 0.50; *p* = 0.62) did not significantly affect OSI values in DMRs. Likewise, the two-way interaction between breeding status and sex (t = 1.49, *p* = 0.15, Fig. 1a) and body mass (t =  − 0.311, *p* = 0.76) did not significantly affect OSI value in DMRs. Similarly, TOS values possessed by DMRs were unaffected by breeding status (t = 1.72, *p* = 0.09), sex (t = 0.26, *p* = 0.80), breeding status*sex (t =  − 0.94, *p* = 0.35, Fig. 1b) and body mass (t = 0.92, *p* = 0.36). Similarly, breeding status (t = 1.80, *p* = 0.07) and body mass (t =  − 0.96, *p* = 0.33) did not significantly affect TAC values in DMRs. However, sex (t = 2.35, *p* = 0.02) did significantly affect TAC; male DMRs (1332.1 ± 89.6 µmol Trolox equivalents/L) possessed higher levels of TAC in comparison to females (1313.0 ± 63.2 µmol Trolox equivalents/L). Although breeding status *sex significantly affected TAC in DMRs (t =  − 2.88, *p* = 0.004, Fig. 1c), post-hoc analyses yielded no significant differences (*p* ≥ 0.08).

### Naked mole-rats

Both the breeding status (t = 2.40, *p* = 0.02) and sex (t = 2.10; *p* = 0.04) did significantly affect OSI values in NMRs. Breeding NMRs (0.97 ± 0.1) possessed higher OSI values than non-breeding NMRs (0.72 ± 0.07); while female NMRs (0.96 ± 0.10) possessed higher OSI values than males (0.72 ± 0.06). Both body mass (t = 0.114, *p* = 0.91) and breeding status*sex (t =  − 1.51, *p* = 0.13, Fig. 1a) did not affect the OSI values of NMRs. Similarly, breeding status did significantly affect TOS (t =  − 2.12, *p* = 0.04), with breeding NMRs (22.1 ± 2.93 µmol H_2_O_2_ equivalent/L) possessing higher TOS values than non-breeding NMRs (13.6 ± 1.70 µmol H_2_O_2_ equivalent/L). However, sex (t =  − 1.58, *p* = 0.13), breeding status*sex (t = 1.34, *p* = 0.20, Fig. 1b) and body mass (t = -0.90, *p* = 0.34) did not affect TOS in NMRs. Lastly, NMRs TAC values were unaffected by breeding status (t =  − 0.82, *p* = 0.42), sex (t =  − 0.25, *p* = 0.81), breeding status*sex (t = 0.39, *p* = 0.70, Fig. 1c) and body mass (t =  − 0.57, *p* = 0.57). Age had no significant correlation with OSI, TOS or TAC in NBF or NBM NMRs (Correlation coefficient ≤ 0.41, q ≥ 0.07, for all).

## Discussion

NMRs and DMRs share many behavioural and physiological traits due to their shared subterranean lifestyle; therefore, it was expected that they would demonstrate similar oxidative stress patterns [35, 66]. Contrasting to expectations, marked differences in oxidative stress patterns within each species were observed. The authors want to highlight the complication of a two species comparison for several reasons such as speciation between species, no genetic exchange, different environmental conditions, with identical selections pressures to be highly unlikely [67]. These factors can all increase the likelihood of a type 1 error, however, since there are only two mammalian species, a multiple species comparison approach cannot be used to circumvent this problem and this problem is unavoidable [67]. In light of this problem the response of each species is discussed and not directly compared. It is, however, important to indicate that NMRs demonstrated much higher oxidative stress in comparison to DMRs, however, this was somewhat expected as NMR have inherently high oxidative damage (TOS) for several reasons [44, 68]. In particular, enzymatic antioxidant activity is accepted to be lower in NMRs, with glutathione peroxidase and cytosolic glutathione-S-transferase levels being lower compared to other species [44]. Lastly, there are female species differences in reproductive output (litter size and frequency of reproduction) [42, 60]. NMR females produce larger litters and have more litters per year which is very likely to contribute to elevated oxidative stress in NMR BFs compared to DMR BFs [35, 69]. Consequently, a direct two-species comparison with the only two eusocial mole-rat species [37] should be avoided due to the factors stated above. Hopefully, as research continues on LRS on the less studied species of social African mole-rats, such as those of the genus *Fukomys* and *Cryptomys*, other species may be as accepted into the eusocial fold thus allowing for increased cross species comparisons.

NMRs demonstrated significant OSI and TOS differences between breeders and non-breeders, with breeding NMR being observed to possess higher TOS and OSI values than non-breeding colony members. Consequently, NMRs follow the classical life-history trade-offs theory, which indicates an inherent cost of reproduction [20, 70]. Furthermore, females were observed to possess higher OSI values than males, however, this is likely driven by high OSI levels in BFs.

NMR BFs ovulate and usually produce usually large litters of offspring throughout the year, again suggesting that oxidative stress is suspected to impart a cost to reproduction [20, 70]. We hypothesise that the mechanism of physiological reproductive suppression, through the use of prolactin, may be the cause of the vast differences between breeders and non-breeders in females and to some extent males [49]. The physiological suppression in NMRs from elevated plasma prolactin levels [49, 50] inherently suppresses reproductive hormones such as oestrogen, progesterone and testosterone [71, 72]. BF NMRs possesses higher levels of both oestrogen, progesterone and testosterone [36, 50]. NMR BMs and BFs possess increased concentration of testosterone compared to their non-breeding counterparts [36, 50]. Oestrogens promote antioxidant enzyme functions [73, 74], where progesterone may antagonise vasoprotective effects of oestrogens [75] and can even amplify oxidative stress [76]. Testosterone can increase susceptibility to ROS through increased metabolism and other pro-oxidative mechanisms contributing to TOS [77]. Furthermore, higher prolactin levels, resulting in hyperprolactinemia has been demonstrated to increase oxidative damage [78–80]. All these factors are likely to contribute to changes in redox balance, however, it should be noted that the higher prolactin levels observed in NMRs may not necessarily cause hyperprolactinemia in this species, and in turn, result in detrimental effects in oxidative stress and requires further investigation.

NMR NBFs and NMR NBMs do not show follicle development or spermatogenesis respectively, thus indicating almost no resource investment into their own reproduction [35, 48]. This physiological difference in reproductive investment and its associated physiological changes may explain the significant difference observed between breeders and non-breeders in addition to the act of breeding. High TAC values were observed in all NMRs which may suggest that NMRs do not just rely on factors that manage post-oxidative damage control such as upregulating NRF2 transcription factors for increased protection [81, 82], tolerating damage to proteins [83, 84] and lipids [85, 86] and upregulated DNA repair pathways [87], but higher reliance on non-enzymatic antioxidant activity.

It is proposed that NMR follow the oxidative theory of ageing and accumulate damage at a very minimal rate [88, 89]. Furthermore, an increase in oxidative stress from reproduction may be negligible in this species and thus may not need to protect themselves pre-emptively from reproductive investment. From this standpoint, pre-emptive mitigation of increased oxidative stress may result in extra resources spent for NMR BFs, and therefore they may not follow the oxidative shielding hypothesis. This is supported by NMRs reproducing even when they are old, with no signs of menopause and they simply deal with the damage [31, 44, 90].

DMRs breeders and non-breeders showed similar OSI and TOS levels but different TAC levels between the sexes: similar plasma TAC levels have been observed in previous studies [33]. Significant increases in TAC but not TOS was observed in the BMs as compared to NBMs which will still lead to BM’s and NBM’s demonstrating similar OSI. A similar pattern was observed for BF’s where TAC was lower and TOS higher compared to NBFs, but not sufficient to result in significant OSI changes between breeders and non-breeders. This is important as OSI is a measure of redox balance derived from the ratio between TAC and TOS.

Damaraland mole-rats employ both physiological and behavioural reproductive suppression and do not display elevated prolactin levels (apart from normal reproductive function from breeders) [49]. It is suggested that the physiological suppression of reproductive hormones is not as strict as seen in NMRs [49], and incest avoidance is the primary driver of reproductive suppression [38]. Additionally, reproductive hormones have been observed to be similar between breeding and non-breeding individuals at varying times of the year [91], which suggest minimal hormonal causes to changes in redox balance between breeders and non-breeders.

Damaraland mole-rats, regardless if they are breeders or non-breeders, invest in reproduction as follicle development is only arrested at the Graafian follicle stage in females and spermatogenesis is not suppressed in males, which suggests that only the act of breeding and the carrying of offspring (i.e. being pregnant) and their associated physiological changes is what separates a breeder and a non-breeder [35, 48, 51]. Previous work supports oxidative shielding in DMRs, where BFs have lower long-term oxidative damage in the liver [33]. The act of breeding results in non-significant redox changes between breeders and non-breeders in DMRs. To date, including this study, long-term oxidative damage markers have not been investigated in NMR BFs. Consequently, there is no clear evidence supporting or opposing the oxidative shielding hypothesis in NMR BFs and require further investigation in the future.

This study provides the first experimental evidence of differences in immediate oxidative stress in two eusocial mole-rat species with similar ecological lifestyles, but different physiological mechanisms and reproductive strategies. This highlights possible divergent mechanisms in dealing with the oxidative cost of reproduction, despite evolving eusociality convergently. At present, this study emphasises the importance of reproductive investment and reproductive strategies to oxidative stress, and future studies should correlate immediate oxidative stress to markers of long-term oxidative damage in these species. Our findings promote that African mole-rats are an ideal group of animals to investigate the reproduction-survival life-history trade-off further. Redox balance results obtained from OSI demonstrated a similar pattern to GSH:GSSG in the liver between the species [44], which suggests the current study methodology to investigate redox balance is not only viable, but may be better suited for research to infer whole-body oxidative stress in smaller and rare animals involved in longitudinal studies and/or for non-destructive sampling.

## Supplementary Information

**Additional file 1: Tables S1 and S2.** Sample details and respective TOS (total oxidant status), TAC (total antioxidant capacity) and OSI (oxidative stress index) for Naked mole-rats and for Damaraland mole-rats. NBF: non-breeding females, NBM: non-breeding males, BM: breeding males, BF: breeding females.

## Data Availability

All data analysed during this study are included in this published article and its supplementary information files.
