# Supplementary material for: Plasma oxidative stress in reproduction of two eusocial African mole-rat species, the naked mole-rat and the Damaraland mole-rat
Source: Front Zool. 2021 Sep 17;18:45. doi: 10.1186/s12983-021-00430-z (PMC8447654; doi:10.1186/s12983-021-00430-z)
Supplement: Supplementary file 1 — Additional file 1: Tables S1 and S2. Sample details and respective TOS (total oxidant status), TAC (total antioxidant capacity) and OSI (oxidative stress index) for Naked mole-rats and for Damaraland mole-rats. NBF: non-breeding females, NBM: non-breeding males, BM: breeding males, BF: breeding females. [file 12983_2021_430_MOESM1_ESM.docx]

**Supplementary Material**

**Supplementary Table S1: Sample details and respective TOS, TAC and OSI for naked mole-rats. NBF: non-breeding females, NBM: non-breeding males, BM: breeding males, BF: breeding female.**

| Animal/sample no. | Sex/status | Body mass (g) | Colony | TOS | TAC | OSI |
| --- | --- | --- | --- | --- | --- | --- |
| 10,1 | BF | 72 | 10 | 29,5491 | 2063,50048 | 1,43199 |
| 10,2 | BM | 42 | 10 | 25,9950 | 3068,48299 | 0,84716 |
| 7,1 | BF | 66 | 7 | 20,4710 | 1579,56112 | 1,29599 |
| 6,1 | BF | 77 | 6 | 21,8640 | 1215,94403 | 1,79811 |
| 6,2 | BM | 32 | 6 | 12,0932 | 1266,29916 | 0,95500 |
| 4,1 | BF | 65 | 4 | 6,9295 | 1107,81300 | 0,62551 |
| 4,2 | BM | 35 | 4 | 19,3098 | 3476,62462 | 0,55542 |
| 3,9 | BF | 75 | 3 | 40,2771 | 4164,63479 | 0,96712 |
| 3,2 | BM | 44 | 3 | 42,6877 | 4185,30690 | 1,01994 |
| 5,8 | BF | 47 | 11 | 36,0202 | 2660,34135 | 1,35397 |
| 6,5 | BM | 33 | 11 | 12,7305 | 1874,27118 | 0,67922 |
| 5,1 | BF | 49 | 5 | 15,2242 | 2719,17736 | 0,55988 |
| 5,2 | BM | 47 | 5 | 9,7935 | 1286,97127 | 0,76097 |
| 1,1 | BF | 71 | 1 | 27,8136 | 2461,04103 | 1,13016 |
| 1,2 | BM | 59 | 1 | 10,7935 | 2131,87745 | 0,50629 |
| 3,4 | NBF | 58 | 3 | 29,7758 | 2435,06838 | 1,22279 |
| 4,4 | NBF | 60 | 4 | 6,0252 | 1315,59419 | 0,45798 |
| 4,8 | NBF | 43 | 4 | 21,7053 | 2320,04664 | 0,93555 |
| 4,9 | NBF | 58 | 4 | 14,1385 | 2024,80653 | 0,69827 |
| 6,3 | NBF | 43 | 6 | 12,6851 | 1390,33181 | 0,91238 |
| 6,7 | NBF | 58 | 6 | 10,7456 | 1688,75225 | 0,63630 |
| 7,8 | NBF | 55 | 7 | 5,5743 | 1288,56143 | 0,43260 |
| 4,5 | NBM | 48 | 4 | 11,2998 | 2416,51648 | 0,46760 |
| 4,7 | NBM | 54 | 4 | 13,8413 | 3371,14386 | 0,41058 |
| 4,10 | NBM | 41 | 4 | 3,9219 | 952,50716 | 0,41175 |
| 4,12 | NBM | 30 | 4 | 12,6423 | 2095,30372 | 0,60336 |
| 6,6 | NBM | 38 | 6 | 15,7028 | 1646,34793 | 0,95379 |
| 6,1 | NBM | 43 | 6 | 15,5819 | 1712,07463 | 0,91012 |
| 6,11 | NBM | 34 | 6 | 13,2066 | 1838,22750 | 0,71844 |
| 6,12 | NBM | 35 | 6 | 16,9270 | 1675,50090 | 1,01026 |

**Supplementary Table S2: Sample details and respective TOS, TAC and OSI for Damaraland mole-rats. NBF: non-breeding females, NBM: non-breeding males, BM: breeding males, BF: breeding female.**

| Animal/sample no. | Sex/status | Body mass (g) | Colony | TOS | TAC | OSI |
| --- | --- | --- | --- | --- | --- | --- |
| 13 | BF | 128 | 1 | 5,3325 | 855,07968 | 0,62363 |
| 14 | BM | 137 | 7 | 2,9446 | 1575,69721 | 0,18688 |
| 15 | BF | 86 | 2 | 5,9673 | 1383,46614 | 0,43133 |
| 16 | BM | 129 | 8 | 6,4811 | 1564,74104 | 0,41420 |
| 17 | BF | 120 | 7 | 5,9597 | 1309,76096 | 0,45502 |
| 18 | BM | 169 | 10 | 7,6952 | 1299,30279 | 0,59226 |
| 19 | BF | 188 | 8 | 3,6423 | 920,31873 | 0,39577 |
| 20 | BM | 184 | 11 | 3,5139 | 1102,58964 | 0,31869 |
| 21 | BF | 140 | 10 | 9,7884 | 897,90837 | 1,09013 |
| 22 | BM | 148 | 12 | 2,9849 | 1112,05179 | 0,26841 |
| 23 | BF | 118 | 11 | 3,2242 | 1438,24701 | 0,22417 |
| 24 | BM | 193 | 13 | 5,7708 | 1711,65339 | 0,33715 |
| 25 | BF | 133 | 12 | 22,5516 | 1536,35458 | 1,46787 |
| 26 | BM | 147 | 13 | 11,3451 | 1872,50996 | 0,60588 |
| 27 | BF | 139 | 13 | 2,8867 | 1121,51394 | 0,25739 |
| 28 | BM | 142 | 15 | 21,9320 | 1577,68924 | 1,39013 |
| 29 | NBF | 109 | 1 | 3,4131 | 1123,50598 | 0,30379 |
| 30 | NBF | 137 | 2 | 3,5970 | 1479,58167 | 0,24311 |
| 31 | NBF | 106 | 3 | 6,1159 | 1564,24303 | 0,39098 |
| 32 | NBF | 88 | 7 | 4,7935 | 1342,13147 | 0,35715 |
| 33 | NBF | 134 | 8 | 3,5693 | 1540,33865 | 0,23172 |
| 34 | NBF | 150 | 10 | 2,7456 | 1400,39841 | 0,19606 |
| 35 | NBF | 121 | 10 | 8,6348 | 1451,69323 | 0,59481 |
| 36 | NBF | 120 | 13 | 7,4736 | 1643,92430 | 0,45462 |
| 37 | NBM | 108 | 1 | 10,3526 | 1579,18327 | 0,65557 |
| 38 | NBM | 146 | 2 | 7,3930 | 1670,31873 | 0,44261 |
| 39 | NBM | 155 | 5 | 6,7406 | 1618,52590 | 0,41646 |
| 40 | NBM | 116 | 7 | 3,5013 | 664,68780 | 0,52675 |
| 41 | NBM | 126 | 8 | 2,8363 | 836,95537 | 0,33888 |
| 42 | NBM | 160 | 10 | 2,7632 | 1146,50694 | 0,24101 |
| 43 | NBM | 197 | 10 | 9,2418 | 1015,05354 | 0,91048 |
| 44 | NBM | 134 | 12 | 6,1310 | 966,81862 | 0,63414 |
